# A challenging case of lumbar vertebral burst fracture with alcohol withdrawal delirium: A case report

**DOI:** 10.1097/MD.0000000000032712

**Published:** 2023-01-20

**Authors:** Keisuke Koizumi, Masashi Uehara, Hiroki Oba, Shota Ikegami, Takayuki Kamanaka, Terue Hatakenaka, Yoshinari Miyaoka, Takuma Fukuzawa, Koji Hayashi, Jun Takahashi

**Affiliations:** a Department of Orthopaedic Surgery, Shinshu University School of Medicine, 3-1-1 Asahi, Matsumoto, Nagano, Japan.

**Keywords:** alcohol withdrawal delirium, thoraco-lumbar spinal fusion, vertebral fracture

## Abstract

Delirium tremens is a symptom of alcohol withdrawal syndrome that occurs 48 to 96 hours after the last drink in 5% of withdrawing patients.

**Methods:**

This report describes the clinical progression of a case of lumbar vertebral burst fracture with alcohol withdrawal delirium that was difficult to manage.

**Results:**

A 47-year-old man was rushed to our hospital complaining of lumbar back pain and numbness in both lower extremities resulting from a 6-m fall during civil engineering work. Computed tomography (CT) revealed a L1 burst fracture with a highly protruding bone fragment in the spinal canal. Magnetic resonance imaging disclosed significant compression of the conus and intramedullary signal changes. We immediately performed posterior spinal fusion and vertebroplasty using instrumentation. On the 4th postoperative day, he became severely agitated, as diagnosed as having delirium tremens related to alcohol withdrawal syndrome, and soon began appropriate medication with diazepam. Although his symptoms persisted until 6 days postoperatively, follow-up CT detected no evidence of screw loosening or breakage.

**Conclusion:**

We encountered a patient with severe delirium tremens developing several days after thoraco-lumbar fusion surgery. Prompt internal fixation successfully treated the spinal injury and prevented neurological damage. It may also be necessary to consider treatment strategies for patients with a background of heavy alcohol consumption in consideration of delirium tremens and other symptoms of alcohol withdrawal.

## 1. Introduction

Alcohol withdrawal syndrome occurs in chronically alcoholic patients with decreased sensitivity of gamma amino butyric acid receptors in the neuroinhibitory system and increased sensitivity of N-methyl-d-aspartate receptors in the neuroexcitatory system. Rapid abstinence from alcohol activates the excitatory system, and excessive excitement and withdrawal symptoms begin within a few hours. Increased sensitivity of the dopamine and noradrenergic pathways also contribute to hallucinations and autonomic excitation.^[1]^ As a symptom of alcohol withdrawal syndrome, delirium tremens appears 48–96 hours after the last drink in 5% of withdrawing patients. Hallucinations, disorientation, tachycardia, hyperthermia, agitation, and sweating occur and may persist for 1–5 days. Even with appropriate treatment, complications such as pneumonia, arrhythmia, and myocardial infarction are associated with a mortality rate of approximately 5%.^[2,3]^

Postoperative alcohol withdrawal is a serious problem in alcohol-dependent patients.^[4]^ Alcohol withdrawal syndrome can produce in-ward delirium tremens, with patients often not responding adequately to high doses of benzodiazepines or opioids.^[5,6]^ Several studies have shown an association between excessive alcohol consumption and a high risk of postoperative complications and prolonged hospitalization.^[7–9]^ We herein describe the clinical details and outcome of a patient with severe delirium tremens following thoracolumbar spinal fusion who was difficult to manage.

## 2. Case presentation

**Figure 1.**
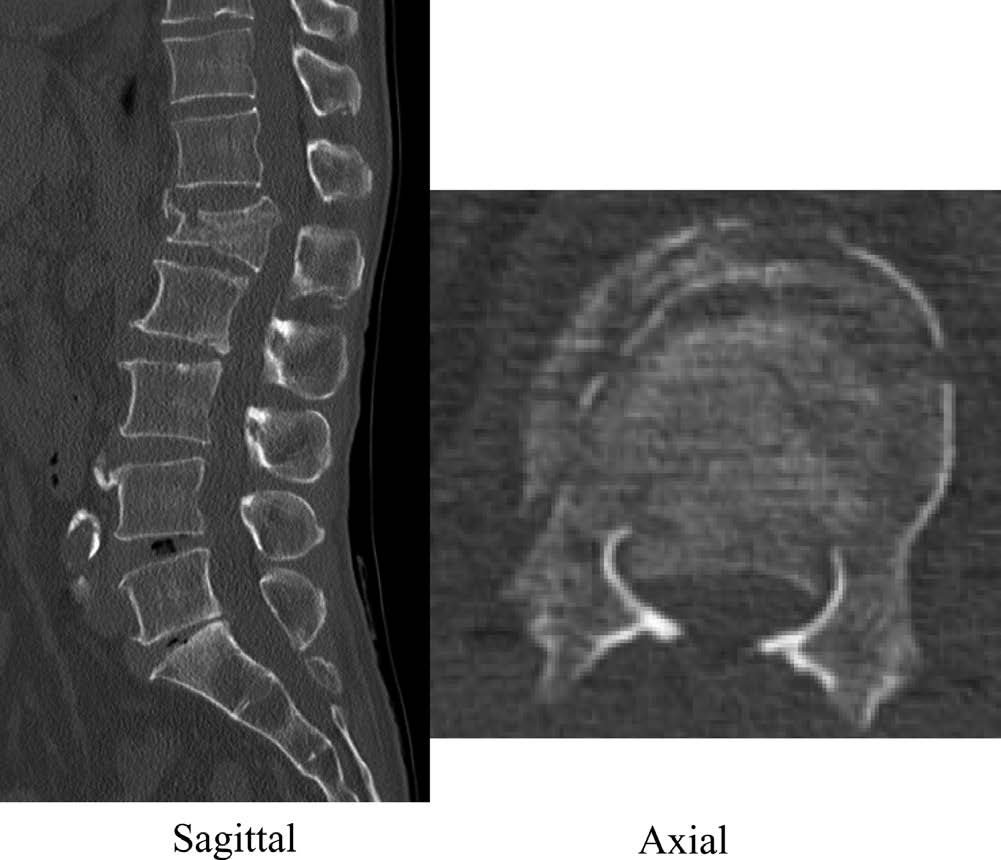
Computed tomography revealed a L1 burst fracture with a highly protruding bone fragment in the spinal canal. The bone fragment occupancy rate was approximately 50%.

**Figure 2.**
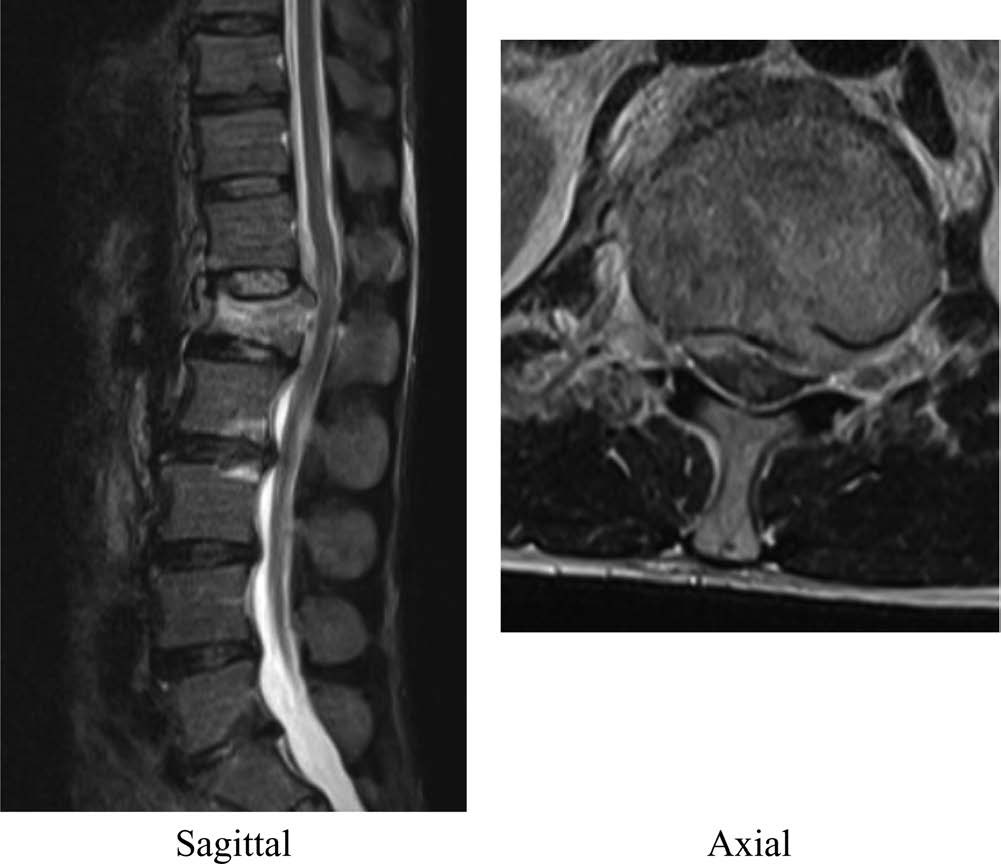
Magnetic resonance imaging showed significant compression of the conus and intramedullary signal changes.

**Figure 3.**
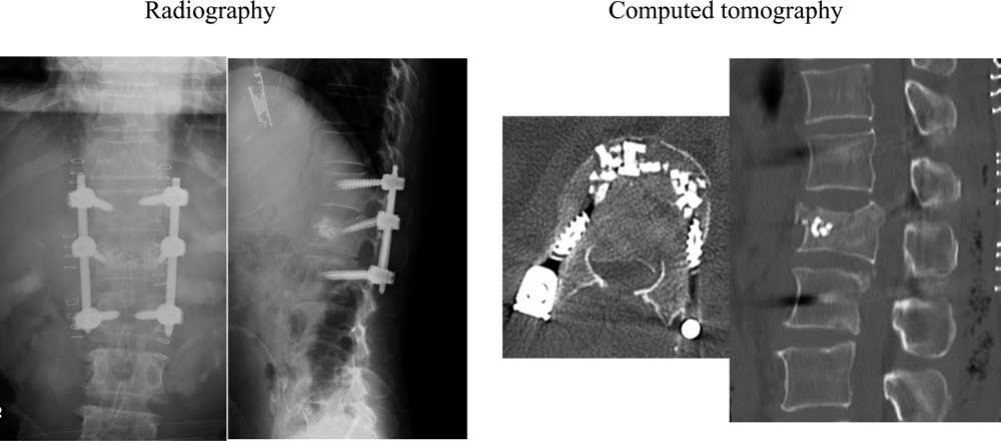
Postoperative radiological findings after spinal fusion and vertebroplasty using instrumentation.

A 47-year-old man was rushed to our hospital complaining of low back pain and numbness in both lower extremities resulting from a fall from a height of 6 m during civil engineering work. His history included 65 grams of ethanol per day of alcohol consumption. Despite his high alcohol intake, he had never been diagnosed as having alcohol dependence syndrome or been hospitalized in relation to drinking. On presentation, his consciousness was clear and his vitals were normal. Muscle assessment revealed preserved muscle strength in the lower extremities. Paresthesia was observed in both lower legs and beyond in addition to contraction of the anal sphincter. A blood test was normal apart from elevations in hepatic enzymes and creatinine kinase. Computed tomography (CT) showed a L1 burst fracture with a highly protruding bone fragment in the spinal canal (Fig. 1). Magnetic resonance imaging revealed significant compression of the conus and intramedullary signal changes (Fig. 2). Posterior spinal fusion and vertebroplasty using instrumentation were performed on the same day (Fig. 3). Postoperatively, we detected no lower limb muscle weakness and improved lower limb numbness. The patient was scheduled to be bedridden at an inclined position of 60 degrees for approximately 3 days until a corset was made, with unrestricted movement in the hospital afterwards.

**Figure 4.**
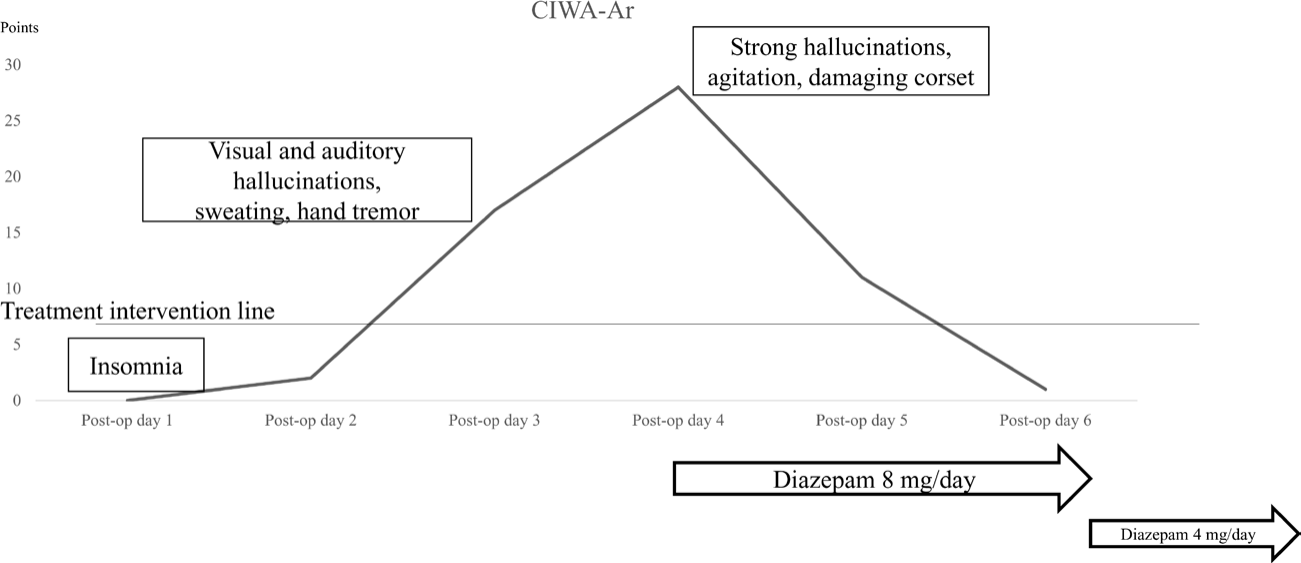
Chronological clinical institute withdrawal assessment for alcohol (CIWA-Ar) score.

On the first postoperative day, he complained of poor sleep (Fig. 4). On the third postoperative day, he displayed symptoms including visual and auditory hallucinations, hand tremors, and sweating. On the fourth postoperative day, he became extremely agitated, pulling on the nurse call button cord, punching the bed light, climbing over the bed rail, failing to comply with bed rest restrictions, and damaging his corset. Consultation with a psychiatrist resulted in a diagnosis of delirium tremens in alcohol withdrawal syndrome and the commencement of appropriate treatment.

**Figure 5.**
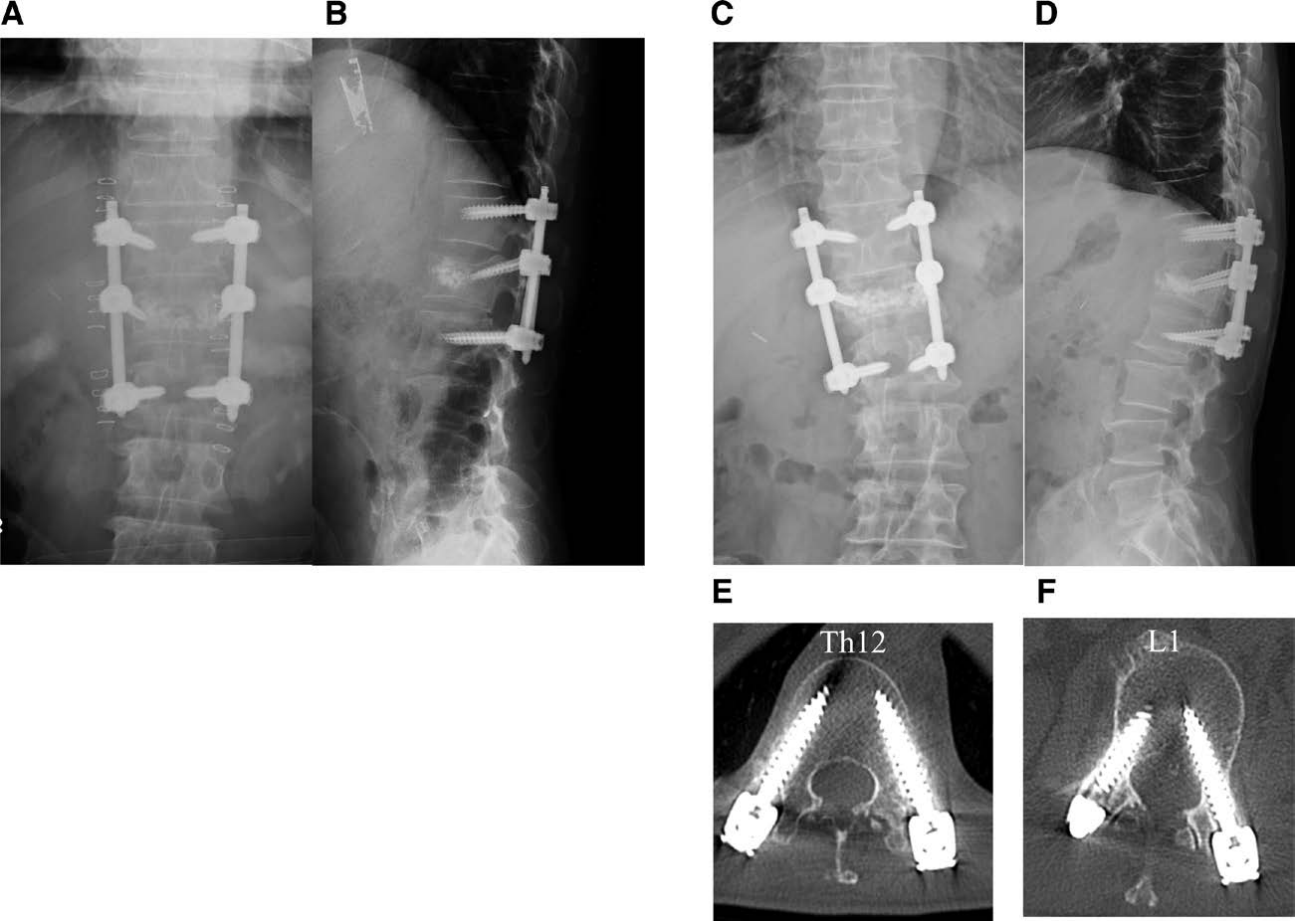
Postoperative radiological findings immediately after surgery (a, b) and 2 months after surgery (c, d, e, and f). No loosening or breakage of screws was detected.

On postoperative day 4, the patient was given diazepam of 8 mg/day for 3 days, followed next by a dose reduction to 4 mg/d for 3 days. Intramuscular diazepam of 10 mg was also administered during strong outbursts. Vitamin B1 was prescribed for preventing Wernicke encephalopathy. On the fifth postoperative day, his visual hallucinations remained, but he became able to communicate with ward staff. He appeared relatively normal on postoperative day 6. Fortunately, follow-up CT showed no evidence of screw loosening or breakage (Fig. 5). He later changed hospitals for further rehabilitation. At 2 months postoperatively, he was walking with a single crutch.

This is a case report. The institutional ethics review board of authors’ facility has confirmed that no ethical approval is required. Consent was obtained from the patient’s parents for participation in this study.

## 3. Discussion

Alcohol withdrawal syndrome is a condition in which the brain’s excitatory system is overactivated by abrupt sobriety in heavy drinkers.^[1]^ Symptoms include minor withdrawal symptoms, visual and auditory hallucinations, withdrawal convulsions, and subsequent delirium tremens, the latter of which occurring in 3–5% of withdrawal syndrome cases.^[2,3]^ As observed in our patient, delirium tremens is characterized by strong hallucinations, disorientation, agitation, sweating, and other delirium symptoms of 1–5 days in duration.^[2,3]^ The time between abstinence from alcohol and the onset of symptoms is relatively long; in the present case, delirium tremens occurred 4 days postoperatively, after the patient had returned to the general ward and his condition stabilized.

There are 2 pillars of alcohol withdrawal management.^[1]^ The first is fixed-dose treatment, in which regular medication dosing is initiated prior to any symptoms in high-risk patients with a history of delirium tremens or withdrawal convulsions. The prediction of alcohol withdrawal severity scale (PAWSS) is considered a useful risk assessment tool in this approach. At a score of ≥ 4, the specificity of PAWSS is 0.93, while a PAWSS score of ≤ 3 has a sensitivity of 0.99.^[10]^ Having a PAWSS score of 0, the reported patient was not a high-risk case and therefore ineligible for fixed-dose treatment. The second withdrawal management method is symptom-based symptom-triggered therapy, which uses the clinical institute withdrawal assessment for alcohol (CIWA-Ar) ring system for quantitatively evaluating the severity of alcohol withdrawal syndrome and recommending intervention at a score of > 9 points.^[11]^ At the onset of delirium tremens in our patient, CIWA-Ar reached 28 points to indicate necessary treatment (Fig. 4).

The patient experienced delirium tremens from the fourth postoperative day after difficulty maintaining a restful sleep and remained in this state for 3 days. If he had been waiting for surgery, he would not have been able to maintain his bed rest limit, which could have resulted in neurological damage due to the progressive bone fragment protrusion in the spine. Prompt surgery halted the progression of the protrusion and spared the patient from neurological deficits. Alcohol withdrawal delirium often occurs even in relatively young patients, and unlike delirium in the elderly, may require additional manpower to treat. Accordingly, patients at risk of delirium should be managed in a well-staffed emergency room or the intensive care unit. Heavy drinkers also tend to suffer fractures even with sufficient bone density, and so bone quality and metabolic calcium/phosphorus balance should be assessed as well. Follow-up mental intervention may be beneficial in the long-term. Although clinicians need to treat immediate problems, such as alcohol withdrawal delirium in this case, there remains the underlying presence of withdrawal syndrome. Additional measures are advocated, including referral to a treatment program in cooperation with a psychiatrist.

## 4. Conclusion

We encountered a challenging case of delirium tremens that developed on the fourth day following thoraco-lumbar spinal fusion. Immediate internal fixation successfully treated the injury and prevented neurological damage. It will also be necessary to consider treatment strategies for patients with a background of heavy alcohol consumption, taking into account dementia tremens and other symptoms of alcohol withdrawal.

## Author contributions

**Conceptualization:** Masashi Uehara.

**Data curation:** Keisuke Koizumi, Masashi Uehara, Hiroki Oba, Shota Ikegami, Terue Hatakenaka, Yoshinari Miyaoka, Takuma Fukuzawa, Koji Hayashi, Jun Takahashi.

**Supervision:** Masashi Uehara, Jun Takahashi.

**Writing – original draft:** Keisuke Koizumi.

**Writing – review and editing:** Masashi Uehara, Hiroki Oba, Shota Ikegami, Terue Hatakenaka, Yoshinari Miyaoka, Takuma Fukuzawa, Koji Hayashi, Jun Takahashi.
